
JOURNAL INFORMATION
==============================
NLM Title Abbreviation: ScientificWorldJournal
Journal ID: ScientificWorldJournal
NLM Journal ID: 101131163
Journal ID: TSWJ

The Scientific World Journal

ISSN: 2356-6140
EISSN: 1537-744X
Publisher: Wiley

ARTICLE INFORMATION
==============================
PMCID: PMC5823147
PMID: 19649512
DOI: 10.1100/tsw.2009.89
Article ID: 832050
Article version: 1
Subjects: Directions in Science

Is Syndecan-2 a Key Angiogenic Element?

Noguer Oriol *
Reina Manuel
Department of Cellular Biology, Faculty of Biology, University of Barcelona, Av.Diagonal 645, 08028, Barcelona, Catalonia, Spain
*Oriol Noguer: onoguer@ub.eduAcademic Editor: Martin Goette

Publication date: 2009
Electronic publication date: 2009 Aug 1
Volume: 9
First page: 729
Last page: 732
Received 2009 Apr 9; Revised 2009 Jun 29; Accepted 2009 Jul 6
Copyright: Copyright © 2009 Oriol Noguer and Manuel Reina.
Copyright year: 2009
License: This is an open access article distributed under the Creative Commons Attribution License, which permits unrestricted use, distribution, and reproduction in any medium, provided the original work is properly cited.
License URL: https://creativecommons.org/licenses/by/3.0/

Keywords: syndecan-2, angiogenesis, migration, endothelial cells, focal adhesions

==============================
Directions in Science
*TheScientificWorldJOURNAL* (2009) 9, 729–732
ISSN 1537-744X; DOI 10.1100/tsw.2009.89

TheScientificWorldJOURNAL
www.thescientificworld.com

### Is Syndecan-2 a Key Angiogenic Element?

O. Noguer* and M. Reina
*Department of Cellular Biology, Faculty of Biology, University of Barcelona, Av. Diagonal 645, 08028, Barcelona, Catalonia, Spain*

**E-mail**: onoguer@ub.edu

*Received April 9, 2009; Revised June 29, 2009; Accepted July 6, 2009; Published August 1, 2009*

**KEYWORDS**: syndecan-2, angiogenesis, migration, endothelial cells, focal adhesions

Angiogenesis is a tightly regulated process in vertebrates that leads to the formation of new blood vessels from pre-existing vessels or by the recruitment of bone marrow–derived endothelial precursor cells[1]. During embryogenesis, after stimulation by proangiogenic factors, such as VEGF or FGF, it contributes to the maturation of the vascular plexus. In adults, it is important in some physiologic conditions, such as wound healing or the reproductive cycle in females, although most of the time it is "switched off" by endogenous inhibitors, such as endostatin or angiostatin. Furthermore, its misregulation is the cause of many pathological situations, as it contributes to tumor development[2], diabetic retinopathy[3], rheumatoid arthritis[4], psoriasis[5], but also cardiovascular disorders[6] and obesity[7].

Although integrins are considered the main matrix adhesion proteins, recent evidence demonstrates an important role for the syndecans in the regulation of this function and for the integration of the extracellular signals from the environment that gives the adequate responses through the modulation of the cytoskeleton[8]. Syndecans are integral transmembrane proteins that belong to the heparan sulfate proteoglycan (HSPG) superfamily. They are known to bind a wide range of ligands, from growth factors to extracellular matrix proteins, due to the high negative charge of the glycosaminoglycan chains they bear[9]. The importance is clear, since all eukaryotic cells express at least one of the four members (syndecans 1–4) described until now. All syndecans share high homology, mainly in the cytoplasmic domain, although they have different ectodomains, which suggests that they have evolved to carry out similar, but nonidentical functions[9,10]. They have been implicated as coreceptors for growth factors[11] in cell-cell[12] and cell-matrix adhesion[13]. However, the involvement of each syndecan in different diseases has only recently been elucidated.

Syndecan-2, identified in fibroblasts, is also the main HSPG expressed by endothelial cells[14]. It has been directly involved in some tumor-related processes[15,16,17], but the most specific function attributed to this proteoglycan is the crucial role that it plays in endogenous fibronectin fibrillogenesis[18], a process involved in the development of fibrotic diseases[19]. Its participation in angiogenesis has also been suggested, since it is essential for new blood vessel sprouting during zebrafish embryogenesis[20] and its down-regulation in mouse endothelial cells reduces the formation of capillary-like structures[21]. In a recent article published in *Experimental Cell Research*, we demonstrated that syndecan-2 plays a central role in many steps of human "*in vitro*" neovascularization[22].

Previous work on syndecan-2 has focused on its intracellular binding partners and its participation in some basic events in different cellular models. It has been involved in adhesion events and actin cytoskeleton organization, as different groups demonstrated the interaction of the cytoplasmic tail of syndecan-2 with proteins such as synbindin[23], syntenin[24], and CASK[25]. In our lab, we have

*Corresponding author.
©2009 with author.
Published by TheScientificWorld; www.thescientificworld.com described the interaction of the PDZ protein Ezrin with the proximal part of the cytoplasmic domain of syndecan-2, and that its overexpression in COS cells induces filopodia through cdc42 activation[26]. We also described how its overexpression in epithelial cells induces a migratory phenotype[27] and stimulates the proliferation in Swiss 3T3 fibroblasts[28]. In addition, other researchers found the same results on colon carcinoma cells[16,17].

In our last study, by down-regulation of syndecan-2 expression with phosphorothioate antisense oligonucleotides in a human endothelial microvascular cell model, we demonstrated its involvement in different cellular functions related to angiogenic processes[22]. We showed that it decreases the spreading and adhesion of these cells when seeded on fibronectin, increased its migration, but impaired the formation of capillary-like structures in matrigel. Additional experiments also demonstrated that the inhibition of this proteoglycan decreased and reorganized the focal adhesions, as shown by paxillin immunostaining, while increasing the stress fibers assembly (Fig. 1). This is consistent with a migratory phenotype, but also with an impairment of the endothelial morphogenesis into tubular structures[29].

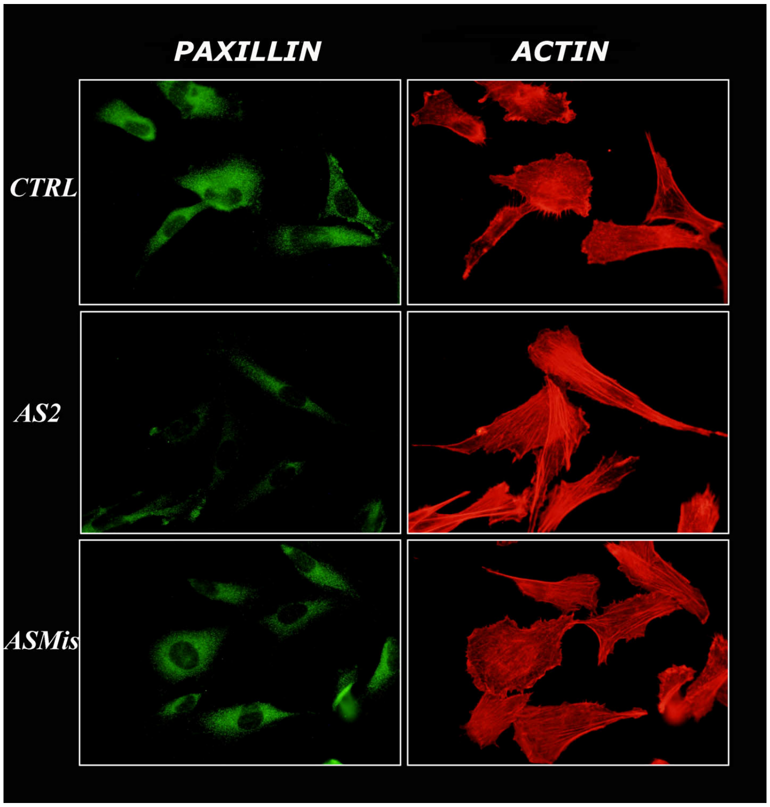

**FIGURE 1.** Syndecan-2 down-regulation redistributed the focal contacts and the actin cytoskeleton. HMEC-1 cells were transfected with the oligonucleotides AS2 and ASMis, and after 24 h in culture, were immunostained with paxillin mAb (focal contacts, *green*) and phalloidin (actin cytoskeleton, *red*). As shown, paxillin staining decreased and reorganized, while the stress fibers became prominent, typical of a migratory phenotype. One of three independent experiments is shown. (n = 3).

We also demonstrated that syndecan-2 protein expression is specifically regulated by well-known proangiogenic growth factors, such as FGF or VEGF, and also different matrices, such as fibronectin or collagen, both in two- or three-dimensional culture conditions. Typically, an enrichment in fibronectin is seen under wound healing conditions, and laminin and collagen deposition has been described for certain tumors[30,31]. As these processes are also characterized by an enhanced release of growth factors and both conditions are able to induce syndecan-2 expression, these proteoglycans may play a pivotal role in giving the adequate response in each situation.

Evidence for integrin and syndecan cooperation in signaling events has become clear in recent years[8], since the regulation of the $\alpha_v\beta_3$ and $\alpha_v\beta_5$ integrins by syndecan-1 during angiogenesis has been demonstrated[32], as well as the interaction with Mac-1[33]. Furthermore, syndecan-2 is able to interact with $\alpha_5\beta_1$ integrin in the regulation of stress fiber formation[34], and with $\alpha_2$ integrin[35] in the control of adhesion and migration. On the other hand, syndecan-2 has also been involved in signaling processes, as in the phosphorylation of FAK[36] or in the activation of src, when this proteoglycan is in complex with p120-GAP[37].

Taking all these considerations together with our results that demonstrated the involvement of syndecan-2 in most of the basic events that take place during neovascularization in endothelial cells cultured "*in vitro*"[22], we propose a role for this proteoglycan as an adaptor protein that is able to integrate different environmental signals essential to induce the angiogenic switch through the regulation of its expression, and a key element in cooperation with the integrins in order to induce cytoskeleton reorganization necessary for capillary morphogenesis.

Although its importance has not been fully elucidated, as it may also be needed in other processes, such as matrix degradation by metalloproteases or in apoptosis, our work helps to clarify the role of this heparan sulfate proteoglycan in the biology of endothelial cells. Further "*in vivo*" experiments may determine if it would be a good target for antiangiogenic therapies, one of the most promising antitumor strategies[38], but also for many other diseases.

### REFERENCES

1. Carmeliet, P. (2003) Angiogenesis in health and disease. *Nat. Med.* **6,** 653–660.
2. Hanahan, D. and Folkman, J. (1996) Patterns and emerging mechanisms of the angiogenic switch during tumorigenesis. *Cell* **86**, 353–364.
3. Gariano, R.F. and Gardner, T.W. (2005) Retinal angiogenesis in development and disease. *Nature* **438**, 960–966.
4. Paleolog, E.M. (2002) Angiogenesis in rheumatoid arthritis. *Arthritis Res.* **4,** S81–90.
5. Detmar, M., Brown, L.F., Claffey, K.P., Yeo, K.T., Kocher, O., Jackman, R.W., Berse, B., and Dvorak, H.F. (1994) Overexpression of vascular permeability factor/vascular endothelial growth factor and its receptors in psoriasis. *J. Exp. Med.* **180,** 1141–1146.
6. Pandya, N.M., Dhalla, N.S., and Santani, D.D. (2006) Angiogenesis - a new target for future therapy. *Vascul. Pharmacol.* **44**, 265–274.
7. Ouchi, N., Kobayashi, H., Kihara, S., Kumada, M., Sato, K., Inoue, T., Funahashi, T., and Walsh, K. (2004) Adiponectin stimulates angiogenesis by promoting cross-talk between AMP-activated protein kinase and Akt signaling in endothelial cells. *J. Biol. Chem.* **279**, 1304–1309.
8. Streuli, C.H. and Akhtar, N. (2009) Signal co-operation between integrins and other receptor systems. *Biochem. J.* **418**, 491–506.
9. Bernfield, M., Götte, M., Park, P.W., Reizes, O., Fitzgerald, M.L., Lincecum, J., and Zako, M. (1999) Functions of cell surface heparan sulfate proteoglycans. *Annu. Rev. Biochem.* **68**, 729–777.
10. Chakravarti, R. and Adams, J.C. (2006) Comparative genomics of the syndecans defines an ancestral genomic context associated with matrilins in vertebrates. *BMC Genomics* **7**, 83.
11. Carey, D.J. (1997) Syndecans: multifunctional cell-surface co-receptors. *Biochem. J.* **327**, 1–16.
12. Leppä, S., Vleminckx, K., Van Roy, F., and Jalkanen, M. (1996) Syndecan-1 expression in mammary epithelial tumor cells is E-cadherin-dependent. *J. Cell Sci.* **109**, 1393–1403.
13. Woods, A. and Couchman, J.R. (1994) Syndecan 4 heparan sulfate proteoglycan is a selectively enriched and widespread focal adhesion component. *Mol. Biol. Cell* **5,** 183–192.
14. Mertens, G., Cassiman, J.J., Van den Berghe, H., Vermylen, J., and David, G. (1992) Cell surface heparan sulfate proteoglycans from human vascular endothelial cells. Core protein characterization and antithrombin III binding properties. *J. Biol. Chem.* **267**, 20435–20443.
15. Han, I., Park, H., and Oh, E.S. (2004) New insights into syndecan-2 expression and tumourigenic activity in colon carcinoma cells. *J. Mol. Histol.* **35**, 319–326.
16. Park, H., Kim, Y., Lim, Y., Han, I., and Oh, E.S. (2002) Syndecan-2 mediates adhesion and proliferation of colon carcinoma cells. *J. Biol. Chem.* **277**, 29730–29736.

17. Kim, Y., Park, H., Lim, Y., Han, I., Kwon, H.J., Woods, A., and Oh, E.S. (2003) Decreased syndecan-2 expression correlates with trichostatin-A induced-morphological changes and reduced tumorigenic activity in colon carcinoma cells. *Oncogene* **22**, 826–830.
18. Klass, C.M., Couchman, J.R., and Woods, A. (2000) Control of extracellular matrix assembly by syndecan-2 proteoglycan. *J. Cell Sci.* **113**, 493–506.
19. Castelletti, F., Donadelli, R., Banterla, F., Hildebrandt, F., Zipfel, P.F., Bresin, E., Otto, E., Skerka, C., Renieri, A., Todeschini, M., Caprioli, J., Caruso, R.M., Artuso, R., Remuzzi, G., and Noris, M. (2008) Mutations in FN1 cause glomerulopathy with fibronectin deposits. *Proc. Natl. Acad. Sci. U. S. A.* **105**, 38–43.
20. Chen, E., Hermanson, S., and Ekker, S. (2004) Syndecan-2 is essential for angiogenic sprouting during zebrafish development. *Blood* **103**, 1710–1719.
21. Fears, C.Y. and Woods, A. (2006) The role of syndecans in disease and wound healing. *Matrix Biol.* **25**, 443–456.
22. Noguer, O., Villena, J., Lorita, J., Vilaró, S., and Reina, M. (2009) Syndecan-2 downregulation impairs angiogenesis in human microvascular endothelial cells. *Exp. Cell Res*. **315**, 795–808.
23. Ethell, I.M., Hagihara, K., Miura, Y., Irie, F., and Yamaguchi, Y. (2000) Synbindin, a novel syndecan-2-binding protein in neuronal dendritic spines. *J. Cell Biol*. **151**, 53–68.
24. Grootjans, J.J., Zimmermann, P., Reekmans, G., Smets, A., Degeest, G., Dürr, J., and David, G. (1997) Syntenin, a PDZ protein that binds syndecan cytoplasmic domains. *Proc. Natl. Acad. Sci. U. S. A.* **94,** 13683–13688.
25. Hsueh, Y.P., Yang, F.C., Kharazia, V., Naisbitt, S., Cohen, A.R., Weinberg, R.J., and Sheng, M. (1998) Direct interaction of CASK/LIN-2 and syndecan heparan sulfate proteoglycan and their overlapping distribution in neuronal synapses. *J. Cell Biol*. **142**, 139–151.
26. Granés, F., Urena, J.M., Rocamora, N., and Vilaró, S. (2000) Ezrin links syndecan-2 to the cytoskeleton. *J. Cell Sci*. **113**, 1267–1276.
27. Contreras, H.R., Fabre, M., Granés, F., Casaroli-Marano, R., Rocamora, N., Herreros, A.G., Reina, M., and Vilaró, S. (2001) Syndecan-2 expression in colorectal cancer-derived HT-29 M6 epithelial cells induces a migratory phenotype. *Biochem. Biophys. Res. Commun*. **286**, 742–751.
28. Villena, J., Berndt, C., Granés, F., Reina, M., and Vilaró, S. (2003) Syndecan-2 expression enhances adhesion and proliferation of stably transfected Swiss 3T3 cells. *Cell Biol. Int*. **27**, 1005–1010.
29. Kanda, S., Miyata, Y., and Kanetake, H. (2004) Role of focal adhesion formation in migration and morphogenesis of endothelial cells. *Cell Signal*. **16**, 1273–1281.
30. Nielsen, G.P., O'Connell, J.X., Wehrli, B.M., and Rosenberg, A.E. (2003) Collagen-rich tumors of soft tissues: an overview. *Adv. Anat. Pathol*. **10**, 179–199.
31. Kosmehl, H., Berndt, A., and Katenkamp, D. (1996) Molecular variants of fibronectin and laminin: structure, physiological occurrence and histopathological aspects. *Virchows Arch*. **429**, 311–322.
32. Beauvais, D.M., Ell, B.J., McWhorter, A.R., and Rapraeger, A.C. (2009) Syndecan-1 regulates alphavbeta3 and alphavbeta5 integrin activation during angiogenesis and is blocked by synstatin, a novel peptide inhibitor. *J. Exp. Med*. **206,** 691–705.
33. Diamond, M.S., Alon, R., Parkos, C.A., Quinn, M.T., and Springer, T.A. (1995) Heparin is an adhesive ligand for the leukocyte integrin Mac-1 (CD11b/CD1). *J. Cell Biol*. **130**, 1473–1482.
34. Kusano, Y., Oguri, K., Nagayasu, Y., Munesue, S., Ishihara, M., Saiki, I., Yonekura, H., Yamamoto, H., and Okayama, M. (2000) Participation of syndecan 2 in the induction of stress fiber formation in cooperation with integrin alpha5beta1: structural characteristics of heparan sulfate chains with avidity to COOH-terminal heparin-binding domain of fibronectin. *Exp. Cell Res.* **256**, 434–444.
35. Choi, S., Kim, Y., Park, H., Han, I.O., Chung, E., Lee, S.Y., Kim, Y.B., Lee J.W., Oh E.S., and Yi, J.Y. (2009) Syndecan-2 overexpression regulates adhesion and migration through cooperation with integrin alpha2. *Biochem. Biophys. Res. Commun*. **384**, 231–235.
36. Park, H., Han, I., Kwon, H.J., and Oh, E.S. (2005) Focal adhesion kinase regulates syndecan-2-mediated tumorigenic activity of HT1080 fibrosarcoma cells. *Cancer Res*. **65**, 9899–9905.
37. Huang, J.W., Chen, C.L., and Chuang, N.N. (2005) P120-GAP associated with syndecan-2 to function as an active switch signal for Src upon transformation with oncogenic ras. *Biochem. Biophys. Res. Commun.* **329**, 855–862.
38. Carmeliet, P. (2005) Angiogenesis in life, disease and medicine. *Nature* **438,** 932–936.

**This article should be cited as follows:**

Noguer, O. and Reina, M. (2009) Is syndecan-2 a key angiogenic element? *TheScientificWorldJOURNAL* **9**, 729–732. DOI 10.1100/tsw.2009.89.
